# Stallion Sperm Viability, as Measured by the Nucleocounter SP-100, Is Affected by Extender and Enhanced by Single Layer Centrifugation

**DOI:** 10.4061/2010/659862

**Published:** 2009-11-12

**Authors:** J. M. Morrell, A. Johannisson, L. Juntilla, K. Rytty, L. Bäckgren, A.-M. Dalin, H. Rodriguez-Martinez

**Affiliations:** ^1^Division of Reproduction, Department of Clinical Sciences, Swedish University of Agricultural Sciences (SLU), P.O. Box 7054, 75007 Uppsala, Sweden; ^2^Department of Anatomy, Physiology & Biochemistry, Swedish University of Agricultural Sciences (SLU), P.O. Box 7011, 75007 Uppsala, Sweden

## Abstract

On-stud assessment of stallion sperm quality can be problematic. A new instrument, the Nucleocounter SP-100, was validated for measuring stallion sperm concentration and viability. It was subsequently used to evaluate sperm viability in Kenney's extender and INRA96. There was a strong correlation between sperm concentrations measured by the Nucleocounter SP-100 and by the Bürker counting chamber (*r* = 0.84; *P* < .001). Similarly, there was a good correlation between sperm viability results from the Nucleocounter SP-100 and flow cytometric results (*r* = 0.73; *P* < .001). Sperm viability at 24 hours was significantly better for samples extended in INRA96 than in Kenney's extender (*P* < .001). Furthermore, sperm kinematics were better for stored samples in INRA96 than in Kenney's extender. Single Layer Centrifugation selected spermatozoa that maintained their viability better during storage for 24 hours than the uncentrifuged samples. In conclusion, the type of semen extender used and Single Layer Centrifugation were found to influence both the kinematics and viability of stallion spermatozoa. The Nucelocounter-SP100 was considered to be a useful instrument for rapidly measuring stallion sperm concentration and viability.

## 1. Introduction

Although many attempts have been made to improve the quality of stallion semen for use in artificial insemination (AI), for example, by changing the composition of semen extenders and by using sophisticated methods of analysis to choose which ejaculates to use, there is still considerable variation in quality among semen doses. The semen extenders traditionally used for stallion semen were variations of the formulation proposed by Kenney et al. [1], but more recent commercially available extenders for stallion semen are based on chemical components rather than on skimmed milk [2–4]. It has been shown that these extenders, which can be described as more chemically defined than Kenney's extender, may increase retention of sperm motility and sperm viability, although the sample size used in the studies was small [2–4].

Determining the quality of a stallion ejaculate for artificial insemination is still considered to be problematical [5]. Several analyses exist which are objective measures of semen quality, for example, sperm motility, morphology, viability, and chromatin integrity, yet no single test has been found to be predictive of likely fertility in the case of stallion semen [5]. Even if all individual stud farms had access to the equipment and skilled personnel required to perform the analyses, there would be insufficient time to analyse each ejaculate before it was required for use. An alternative approach would be to select the most robust spermatozoa from the ejaculate, for example, by Single Layer Centrifugation (SLC) [6–8]. In this method, spermatozoa are centrifuged through a column (single layer) of glycidoxypropyltrimethoxysilane-coated silica in a species-specific formulation (Androcoll*™*), resulting in the selection of motile, morphologically normal spermatozoa with intact membranes and good chromatin integrity [7, 8]. 

One analysis which could be of use in evaluating stallion ejaculates is sperm viability or, more accurately, sperm membrane integrity. The assay determines penetration of fluorescent dye into spermatozoa through porous membranes or its exclusion from spermatozoa with intact membranes. Typically, the proportion of spermatozoa excluding dye can be counted either by fluorescence microscopy [9] or by flow cytometry [9, 10]. Fluorescence microscopy is time-consuming, involving the subjective evaluation of each spermatozoon, whereas flow cytometry is objective and can rapidly measure thousands of spermatozoa. However, the flow cytometer is expensive and the staining technique too time-consuming to be useful for field use.

Recently a new instrument has been used to evaluate boar sperm concentration [11] and both the concentration and viability of hybridoma cells [12]. Due to its compact size and its relatively inexpensive purchase price (approximately 13000 Euros in 2008), this instrument could be useful for field measurements of both concentration and viability. The objectives of this study were to (i) evaluate the Nucleocounter SP-100 for measuring stallion sperm concentration and viability; (ii) use the Nucleocounter SP-100 to evaluate stallion sperm viability in two semen extenders, Kenney's extender and INRA96; (iii) to investigate the effect of Single Layer Centrifugation on stallion sperm viability.

## 2. Materials and Methods

### 2.1. Animals and Semen Collection

Warmblood stallions of breeding age and documented fertility were housed under standard husbandry conditions at two commercial studs in Sweden, Markebäck (3 stallions, 10–19 years old, mean age 13.3 years) and Flyinge AB (12 stallions, 4–25 years, mean age 8.7 years). At Markebäck, semen was collected three times during one week on alternate days. At Flyinge, semen was collected up to three times a week during the normal breeding season, as part of the usual semen collection routine of the stud. The stallions mounted a phantom and ejaculated into a warmed artificial vagina (Colorado or Missouri type, depending on which worked best for each individual), the semen being collected into a warm plastic bottle fitted with a filter to capture gel. Aliquots (10–15 mL) of the gel-free portion of the ejaculates were made available for the experiments. The remainder of the ejaculates collected at Flyinge was processed and supplied to customers for AI according to their usual procedure. In all cases, the aliquots for the experiment were removed immediately from the rest of the ejaculate and were extended 1 : 1 (v/v) with extender at 37°C (see experimental design). Ejaculates collected at Flyinge were processed immediately; those collected at Markebäck were extended with INRA96, drawn into standard syringes used for AI doses, placed in a styrofoam insulated box at room temperature (approximately 20–22°C), and transported by car to the laboratory at SLU, for further processing approximately 5 hours after collection.

### 2.2. Media

(Kenney's extender [1]) To 100 mL distilled water were added glucose (4.9 g), skimmed milk powder (2.4 g), dihydrostreptomycin (0.15 g), and penicillin (sodium salt) (0.15 g). The glucose and skimmed milk powder were purchased from Sigma, Stockholm, Sweden, and the antibiotics from Apoteket, Stockholm, Sweden. INRA96 was purchased from IMV.

### 2.3. Sperm Concentration

Aliquots of extended semen (100 *μ*L) were diluted 1 : 99 with 3% phosphate buffered saline (PBS) for sperm counting in a Bürker chamber. The same observer performed all the counting, to provide continuity; each sample was counted once.

### 2.4. Single Layer Centrifugation

The method was described previously [13]. Briefly, 4 mL Androcoll*™*-E (patent applied for) were pipetted into a centrifuge tube and an aliquot of approximately 450 million spermatozoa (4.5 mL of extended semen containing approximately 100 × 10^6^ spermatozoa/mL) was layered on top. After centrifugation at 300 *x*g for 20 minutes, the supernatant and most of the colloid was discarded and the sperm pellet was transferred to a clean centrifuge tube containing 3 mL extender. No liquid seminal plasma was transferred with the sperm pellet.

### 2.5. Subjective Estimation of Motility

Aliquots (5.0 *μ*L) of the extended ejaculate were examined by phase contrast light microscopy (*x* 400) using a heated microscope stage (38°C), immediately after preparation and once daily until the motility had dropped to 20%. Approximately 200 spermatozoa in several fields were examined on each occasion. One observer, who was unaware of the identity of the samples, performed all motility assessments. Between observations, the samples were stored in a refrigerator at 6°C in an insulated holder (see experimental design) and were allowed to equilibrate to room temperature for approximately 15 minutes before motility assessment on the heated stage.

### 2.6. Computer-Assisted Sperm Analysis (CASA)

At SLU, CASA was performed on 5 *μ*L aliquots of sperm samples placed in a prewarmed Makler chamber (Sefi Medical Instruments, Haifa, Israel), depth 10 *μ*m, using a Mika Cell Motion Analyzer (MTM Medical Technologies Montreux, Switzerland). Sperm motility was assessed in a microscope equipped with a warm stage and phase contrast optics (20x objective, Optiphot-2, Nikon, Japan). For each sample, a total of 200 spermatozoa were assessed. The settings had been previously determined to be optimal for stallion spermatozoa [14]. The following parameters were measured: motility (mot, %), curvilinear velocity (VCL, *μ*m/s), straight line velocity (VSL, *μ*m/s), velocity of the smoothed path (VAP, *μ*m/s), and amplitude of lateral head deviation (LHD *μ*m).

### 2.7. Objective Motility Analysis Using the Qualisperm*™* Motility Analyzer

At Flyinge AB, aliquots of the sperm preparations were assessed on a heated stage (38°C) by the Qualisperm*™* motility analyser immediately after extension [15]. In addition, measurements were made after 24 hours on the stored samples (see experimental design). The Qualisperm*™* analyses consisted of total sperm motility (%), progressive motility (%), and mean speed (*μ*m/s).

### 2.8. Sperm Viability Using the Flow Cytometer

The method was as described previously [16]. A combination of the fluorochromes SYBR-14 and propidium iodide (PI) (Live-Dead Sperm Viability Lit L-7011; Invitrogen, Eugene, OR, USA) was used. Fresh sperm samples (500 *μ*L) were extended in 2500 *μ*L Cellwash (Becton Dickinson) and centrifuged for 10 minutes at 400*x*g. The sample was resuspended with 1 mL Cellwash and an aliquot of 300 *μ*L was used for viability assessment. To this aliquot, 1.5 *μ*L SYBR-14 stock solution (1 : 50 in Cellwash) and 1.5 *μ*L PI were added before incubating the mixture for 10 minutes at 37°C. FCM analysis was performed using an LSR flow cytometer (Becton Dickinson), equipped with standard optics. From each sample, a total of 10 000 events were collected and quantified as percentages. Three categories of spermatozoa could be described: live spermatozoa with an intact membrane (SYBR-14+/PI−), moribund (SYBR-14 intermediate/PI intermediate) and dead (SYBR-14+/PI+), according to the degree of intactness of the plasma membrane.

### 2.9. Nucleocounter SP-100

The Nucleocounter SP-100 (Chemometec, Denmark) was used to analyse sperm concentration and sperm viability according to the manufacturer's instructions [9]. For sperm concentration, an aliquot (50 *μ*L) of each sample was diluted with 5 mL reagent S100 and, after mixing, was loaded into a cassette containing propidium iodide. The cassette was inserted into the fluorescence detector and the total number of cells in the sample was reported (T, million). For sperm viability, a further 50 *μ*L-aliquot of the sample was diluted with phosphate buffered saline (5 mL), pH 7.1, supplied by the same manufacturer (Chemometec, Denmark), before loading into another cassette and inserting into the fluorescence detector. This time the instrument reported the number of non-viable cells (N, million). The viable count was determined by subtracting the non-viable cells from the total number of cells (T-N) and expressing the result as a percentage of the total number of cells.

### 2.10. Experimental Design

**Experiment 1**

Aliquots of ejaculates from 3 stallions were made available for viability measurement using the flow cytometer (staining with SYBR 14/PI) and the Nucleocounter SP-100, both on the day of collection (*n* = 24) and after 24 hours (*n* = 36). In addition, two samples were measured repeatedly in the Nucleocounter SP-100 (10 times each) to measure the variability between assays. These samples were selected to represent high and low sperm concentrations and low and high viabilities, respectively.

**Experiment 2**

Forty five semen samples were available (3 from each of 3 stallions and 4 from each of 9 stallions). Each sample was split, with one aliquot being extended with Kenney's extender and the other with INRA96, to give 90 sperm samples altogether. The sperm concentration was adjusted to approximately 100 × 10^6^ spermatozoa per mL in each aliquot. The following parameters were measured for all sperm samples: sperm concentration using the Bürker chamber, concentration and viability with the Nucleocounter SP-100, and sperm motility (subjective assessment and Qualisperm*™* evaluation). The measurements of sperm concentration and viability were repeated on all 90 samples after overnight storage at 6°C in the refrigerator, to give a reading at approximately 24 hours after semen collection.

### 2.11. Statistics

The statistical analyses were performed using the statistical software in the Excell package (Version 2003, Microsoft Corporation, Redmond, WA, USA). Analysis of variance [17] was performed on mean values of all of the parameters assayed at the different time intervals. Correlation and regression analyses [17] were performed on the results for the different methods of assessing concentration and viability. In all cases, significance was set at *P* < .05 level. The correlation between motility and viability was also analysed.

## 3. Results

**Experiment 1**

**Figure 1:**
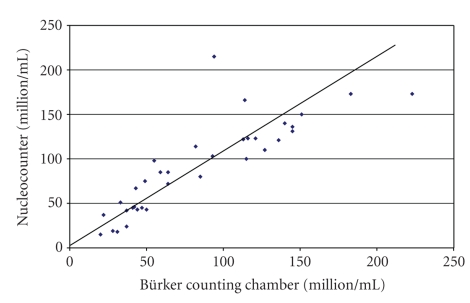
Relationship between sperm concentration measured with the Nucleocounter SP-100 and with the Bürker counting chamber (*n* = 35).

The results for sperm concentration measured by the Nucleocounter SP-100 and the Bürker chamber were highly correlated, as shown in Figure 1 (*r* = 0.85; *P* < .001). Regression analysis showed a highly significant relationship between the two methods (*R* ^2^ = 0.78, *P* < .001).

**Figure 2:**
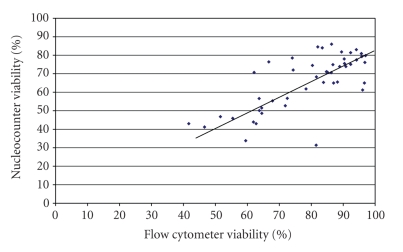
Comparison of viability results using the flow cytometer and the Nucleocounter SP-100 (*n* = 60).

The viability results for the Nucleocounter SP-100 and flow cytometer (Figure 2) were also well correlated (*r* = 0.73,  *P* < .001), although the absolute values were not always similar. Regression analysis showed a significant relationship between the two methods (*R* ^2^ = 0.54, *P* < .001). Repeated readings of the same samples gave values for sperm concentration (mean ± SD) of 195 ± 15 × 10^6^/mL (range 176.2–221.8 × 10^6^/mL) and 75 ± 4 × 10^6^/mL (range 68–80 × 10^6^/mL), respectively, for the high and low concentration samples, and viabilities of 25.2 ± 4.8% (range 17.9–31.4%) and 73.2 ± 3.2% (range 67.5–77.3%) for the low and high viability samples.

When both sets of viability results were compared with CASA motility results, the correlation was significant (*P* < .001) although the correlation was 0.66 and 0.67 for the FC and Nucleocounter SP-100, respectively. However, when the subjective motility results were used there was a stronger correlation between the FC viability results and subjective motility (*r* = 0.82) than for the Nucleocounter SP-100 results and subjective motility (*r* = 0.63). Both correlations were significant (*P* < .001).

**Experiment 2**

**Figure 3:**
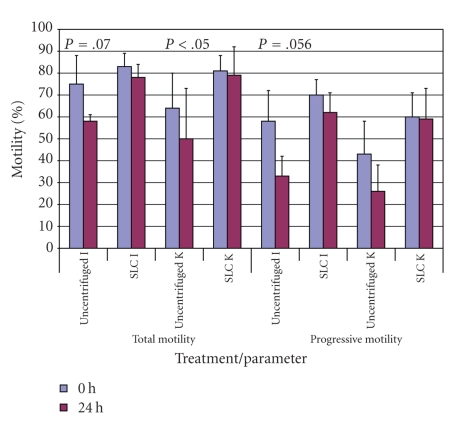
Mean values for total motility (%) and progressive motility (%) for one stallion (Stallion I) (*n* = 4). Note: SLC: Single Layer Centrifugation; I: INRA96; K: Kenney's extender. Significant differences and trends towards significance between 0 hour and 24 hours within each treatment group are shown on the figure. Other significant differences were found as follows: for progressive motility between K uncentrifuged and K SLC at 24 hours (*P* < .05), between I uncentrifuged and I SLC at 24 hours (*P* < .001), and between K SLC and I SLC at 0 hour (*P* < .05); for total motility, between K uncentrifuged and K SLC at 24 hours (*P* < .05), and between I uncentrifuged and I SLC at 24 hours (*P* < .01). There were no differences between ejaculates except for total motility, uncentrifuged, I versus K at 0 hour.

The motility results for unselected and selected spermatozoa were extender and time dependent. Although there were no differences between any of the parameters for uncentrifuged samples at time 0 hour, there were significant differences at 24 hours, with a higher mean total motility (*P* < .05), progressive motility (*P* < .001), and higher velocity (*P* < .001) being observed for the uncentrifuged samples in INRA96 than in Kenney's extender (Table 1). The mean values for the kinematics of SLC-selected spermatozoa were better than the unselected ones for samples in INRA96 at both time points (*P* < .001), but were only significant at 24 hours for samples in Kenney's extender. The mean kinematic values for the ejaculates from one stallion are shown in Figure 3 as an example.

**Figure 4:**
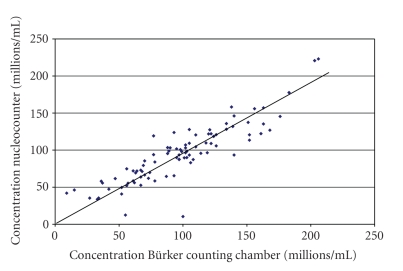
Relationship between sperm concentration measured with the Nucleocounter SP-100 and with the Bürker counting chamber (*n* = 89).

As in the previous experiment, the results for sperm concentration measured by the Nucleocounter SP-100 and the Bürker counting chamber (Figure 4) were closely correlated (*r* = 0.84; *P* < .001).

**Figure 5:**
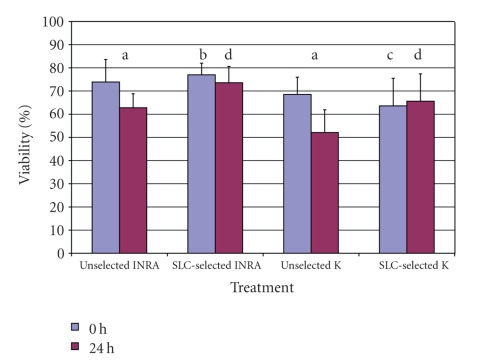
Effect of Single Layer Centrifugation through Androcoll*™*-E on mean stallion sperm viability (*n* = 45). Note: a:difference in sperm viability at 0 and 24 hours was statistically significant for the uncentrifuged samples in both extenders; b: SLC-samples in INRA96 had a higher viability than non-SLC samples at 0 hour (*P* < .001); c: difference between uncentrifuged and SLC-samples in Kenney's extender at 0 hour, *P* < .02; d: SLC samples in both extenders had a higher viability than the uncentrifuged samples at 24 hours (*P* < .001).

Mean values for sperm viability in the different extenders had decreased by 24 hours for the uncentrifuged samples, regardless of extender (*P* < .001), and there was a higher proportion of viable spermatozoa in the SLC groups than in the non-SLC samples at 24 hours (Figure 5). The SLC-selected samples in INRA had better viability than those in Kenney's extender at both time points (0 hour, 77.0 ± 5% versus 63.6 ± 11.9%; 24 hours, 73.6 ± 7.2% versus 65.6 ± 5.6%; *P* < .001). Moreover, sperm viability at 24 hours was significantly better for the samples extended in INRA96 than for those in Kenney's extender (*P* < .001). The differences between stallions (Table 2) were also significant (*P* < .001).

## 4. Discussion

The purpose of this study was twofold: to validate the Nucleocounter SP-100 for measuring stallion sperm concentration and viability, and to use the Nucleocounter SP-100 (as well as sperm kinematics) to investigate the effect of SLC and different semen extenders on stallion sperm survival. The results of the validation show that the Nucleocounter SP-100 gave similar results to sperm concentrations obtained from the Bürker counting chamber, and that the two sets of results were highly correlated. These findings are similar to those of other authors [11, 12], although the former study compared boar semen samples with concentrations between 150 and 1000 million/mL measured by a haemocytometer and the Nucleocounter SP-100 and the latter study measured hybridoma cell suspensions rather than sperm suspensions. Our results for stallion sperm concentration are also in agreement with those reported in a conference abstract for stallion spermatozoa, which showed a highly significant relationship between the two methods [18]. 

For sperm viability, however, the values obtained from FC and Nucleocounter SP-100 were not always similar, although there was a significant correlation between the two sets of results. In the instructions accompanying the Nucleocounter SP-100, it is stated that the dilution medium may have a minor effect on the non-viable count, with the result that the non-viable count may be slightly higher in phosphate buffered saline than in many extenders. Thus it was not unexpected that the viability was slightly lower when measured in the Nucleocounter SP-100 than in the flow cytometer in the experiment reported here. On the other hand, the correlation between CASA motility and Nucleocounter viability was lower than expected, although the relationship was still statistically significant, and a similar correlation was observed between the CASA motility and FC viability results. A higher correlation might have been expected between these objective measurements. However, only 200 spermatozoa were analysed by CASA, which may not have been sufficient in view of the better correlation observed between subjective motility and FC viability. Results from our more recent studies using motility analysis of much higher numbers of spermatozoa are consistently showing that the correlation between motility and viability (measured by the Nucleocounter SP-100) of SLC-selected spermatozoa is not as strong as for uncentrifuged spermatozoa (Morrell et al., unpublished data). One explanation for this observation could be that the lack of seminal plasma in the SLC-selected samples affects sperm kinematics more than it affects their ability to exclude dye in the short term.

The Nucleocounter SP-100 was considered to be user-friendly and rapid, with the measurements for concentration and viability for 12 samples being completed in less than 30 minutes. These observations are comparable with those of [12] who reported an analysis time of about one minute when measuring the viability of cell suspensions. In the current study, the operator estimated that counting the sperm concentrations of 12 samples in the Bürker counting chamber would take approximately 1 hour when done consecutively that is, the sperm preparation in one chamber is being allowed to settle while the previous preparation is being counted. Thus the Nucleocounter SP-100 represents a considerable saving in time over the Bürker counting chamber while giving similar results. A disadvantage is the cost of the cassettes, at 4.0 *€* for each viability test (two casettes). In contrast, FC is not practical for single samples because of the time taken to set up the instrument at the start of the analysis, whereas the Nucleocounter SP-100 would be ideally suited for individual measurements. 

When different semen extenders were compared, INRA96 was better than Kenney's extender for maintaining sperm viability and progressive motility in cold storage. Furthermore, the SLC samples had better sperm kinematics at both time points and retained their viability better than the unselected samples, although there were some differences between extenders. These results are consistent with the previous studies on sperm kinematics using SLC-selected sperm samples [15] and with a preliminary study in which viability of SLC-selected samples was measured by SYBR/PI staining and analysed by flow cytometry [16]. Morphological evaluation, performed on the uncentrifuged and SLC-selected samples from one ejaculate per stallion showed an increase in the proportion of morphologically normal spermatozoa in the SLC-selected samples (data not shown). Again, this result is in agreement with our previous studies [8].

It was surprising that the SLC-selected spermatozoa in Kenney's extender had a lower viability (i.e., ability to exclude dye) than the unselected samples, according to the results from the Nucleocounter SP-100, which is in contrast to our previous observation with SYBR/PI staining and flow cytometric analysis [16]. Equally inexplicable is the apparent increase in viability of these SLC-selected samples after cool storage for 24 hours. However, previous results with Annexin V/propidium iodide suggested that the membranes of SLC-selected spermatozoa resuspended in Kenney's extender may be temporarily unstable, although this instability disappears with time [16]. Therefore, the results reported here with PI staining may be in keeping with these previous observations with Annexin V/PI staining. The previous results in Kenney's extender [16], and also the present results with samples in INRA96, showed that sperm viability was better in the SLC-selected samples and that this viability was retained during storage, in contrast to unselected samples where sperm viability deteriorated with storage. It is not certain whether the temperature of the sample could affect fluorescence detection by the Nucleocounter SP-100, since the viabilities at 0 hour were done on samples at room temperature whilst those at 24 hours were made on samples taken from the refrigerator, during their equilibration for motility analysis. The results of morphological evaluation, although not presented here, showed that the proportion of morphologically normal spermatozoa was higher in the SLC-selected samples than in the unselected samples, in keeping with our previous extensive morphological studies [8, 13].

AI was not a part of the current experiment, although the pregnancy results following AI with the same ejaculates were made available by the stud farm personnel. There was only a low, nonsignificant, correlation between the pregnancy rate following AI and the sperm viability in INRA96 (*r* = 0.375; *P* < .05), although the sample size was small. In contrast, there was a significant correlation between progressive motility (as measured by the Qualisperm*™* Motility Analyser) and pregnancy rate for AI with fresh sperm doses (*r* = 0.6; *P* < .05), and between sperm velocity and pregnancy rate for either fresh or stored insemination doses (*r* = 0.63; *P* < .005).

In conclusion, the Nucleocounter SP-100 was a useful instrument for measuring stallion sperm concentration rapidly and proved to be convenient for measuring sperm viability. However, some further studies are needed concerning the use of this instrument for measuring the viability of stallion spermatozoa. The type of semen extender was found to influence both sperm kinematics and viability. Some parameters of sperm kinematics may be useful in predicting the likely outcome of AI, although viability measurements were not indicative in the small sample used for this study. The most robust spermatozoa were selected by SLC, confirming our previous results.

## Figures and Tables

**Table 1:** Mean (±SD) values for sperm kinematics of cool-stored stallion spermatozoa in different extenders after Single Layer Centrifugation (*n* = 45).

Parameter	Time	Uncentrifuged I	Centrifuged I	Uncentrifuged K	Centrifuged K
Total motility (%)	0 hour	76.4 ± 12.4^b^	83.8 ± 8.9^b^	74.8 ± 12.4	77.1 ± 14.3
	24 hours	63.0 ± 15.5^ab^	78.9 ± 13.9^b^	56.4 ± 17.2^ac^	65.1 ± 19.7^c^

Progressive motility (%)	0 hour	54.7 ± 17^b^	67.3 ± 16.5^b^	52.6 ± 15.7	56.8 ± 18.2
	24 hours	40.2 ± 16^ab^	64.5 ± 16.7^b^	30.8 ± 12.5^ac^	42.7 ± 21^c^

Velocity (*μ*m/s)	0 hours	48.3 ± 14.8^b^	43.7 ± 14.2^b^	46.3 ± 14	40.6 ± 13
	24 hours	38.1 ± 15.6^ab^	49.6 ± 14.5^b^	28 ± 11.5^ac^	36.9 ± 16.5^c^

Note: I:INRA96, K: Kenney extender. a: significant difference between INRA96 and Kenney's extender for uncentrifuged samples; b: significant difference between uncentrifuged and centrifuged samples in INRA96; c: significant difference between uncentrifuged and centrifuged samples in Kenney's extender.

**Table 2:** Variation between stallions in sperm viability (%) in two semen extenders, Kenney's extender and INRA96 (*n* = 12 stallions, 45 ejaculates).

Stallion (no. ejaculates)	Kenney's extender		INRA96	
	0 hour	24 hours	0 hour	24 hours
AA (4)	59 ± 8^a^	43 ± 5^a^	68 ± 4^b^	47 ± 4^b^
DD (4)	70 ± 3^b^	52 ± 4^bd^	74 ± 9	63 ± 7^d^
I (4)	74 ± 3^a^	49 ± 15^ad^	76 ± 5	55 ± 25^d^
J (3)	66 ± 2	60 ± 8^d^	74 ± 2^c^	62 ± 3^cd^
K (4)	69 ± 9^a^	52 ± 6^ad^	71 ± 5	63 ± 6^d^
L (3)	74 ± 6	63 ± 6	81 ± 6	77 ± 7
N (4)	67 ± 4^ae^	58 ± 5^ad^	78 ± 3^ae^	69 ± 4^ad^
O (4)	67 ± 13	54 ± 3^e^	82 ± 4^a^	71 ± 6^ae^
Q (4)	72 ± 7^a^	54 ± 10^ad^	65 ± 27	71 ± 7^d^
R (4)	76 ± 5^c^	47 ± 13^cd^	77 ± 3^a^	63 ± 9^ad^
T (3)	64 ± 5	40 ± 15	69 ± 7	56 ± 13
U (4)	64 ± 5	56 ± 6	72 ± 4	61 ± 10

Note: overall differences between stallions *P* < .001; overall difference between treatments, *P* < .001; overall difference between 0 and 24 hours, *P* < .001.

a: difference between 0 and 24 hours within extender, *P* < .05; b: difference between 0 and 24 hours within extender, *P* < .001; c: difference between 0 hour and 24 hours within extender, *P* < .01; d: difference between extenders, *P* < .05; e: difference between extenders *P* < .01.
